# Bis(μ-propan-2-olato-κ^2^ *O*:*O*)bis­[chlor­ido(propan-2-ol-κ*O*)bis­(pro­pan-1-ol­ato-κ*O*)tin(IV)]

**DOI:** 10.1107/S1600536812007799

**Published:** 2012-02-24

**Authors:** Nikolai Klishin, Oleksii Brusylovets, Anatoliy Brusilovets, Eduard Rusanov

**Affiliations:** a Kiev National Taras Shevchenko University, Department of Chemistry, Volodymyrska Street 64, 01601 Kiev, Ukraine; b Institute of Organic Chemistry, National Academy of Sciences of Ukraine, Chervonatkatska Street 60, 02660 Kiev, Ukraine

## Abstract

The binuclear centrosymmetric title compound, [Sn_2_(C_3_H_7_O)_6_Cl_2_(C_3_H_8_O)_2_], exhibits an edge-shared double octahedral exhibits an edge-shared octa­hedral structure, which is distorted owing to the presence of asymmetric intra­molecular hydrogen bonds between the axially coordinated isopropanol and isopropoxide ligands. The H atom of the hy­droxy group is located nearer to an isoprop­oxy group with the longest Sn—O bond [2.1789 (17) Å].

## Related literature

For the synthesis of the title compound, see: Mehrotra & Gupta (1966 ▶). For related structures, see: Chandler *et al.* (1995 ▶); Genge *et al.* (1996 ▶); Hampden-Smith *et al.* (1991 ▶); Reuter & Kremser (1991 ▶, 1993 ▶); Reuter & Schröder (1992 ▶); Sterr & Mattes (1963 ▶); Webster & Collins (1974 ▶); Zhang *et al.* (2011 ▶). For alcohol adducts of alkoxides, see: Vaartstra *et al.* (1990 ▶).
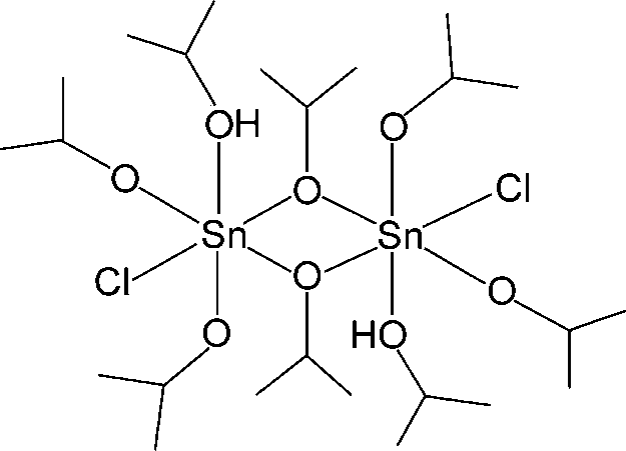


## Experimental

### 

#### Crystal data

[Sn_2_(C_3_H_7_O)_6_Cl_2_(C_3_H_8_O)_2_]
*M* *_r_* = 783.02
Monoclinic, 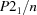

*a* = 11.184 (2) Å
*b* = 10.354 (2) Å
*c* = 15.426 (3) Å
β = 102.19 (3)°
*V* = 1746.0 (6) Å^3^
*Z* = 2
Mo *K*α radiation
μ = 1.62 mm^−1^
*T* = 173 K
0.50 × 0.35 × 0.29 mm

#### Data collection

Nonius KappaCCD diffractometer
Absorption correction: multi-scan (*DENZO*/*SCALEPACK*; Otwinowski & Minor, 1997 ▶) *T*_min_ = 0.498, *T*_max_ = 0.651
16149 measured reflections
3539 independent reflections
3075 reflections with *I* > 2σ(*I*)
*R*_int_ = 0.029

#### Refinement

*R*[*F* ^2^ > 2σ(*F* ^2^)] = 0.021
*wR*(*F* ^2^) = 0.045
*S* = 1.03
3539 reflections
175 parameters
H atoms treated by a mixture of independent and constrained refinement
Δρ_max_ = 0.45 e Å^−3^
Δρ_min_ = −0.32 e Å^−3^

### 

Data collection: *COLLECT* (Nonius, 1998 ▶); cell refinement: *DENZO*/*SCALEPACK* (Otwinowski & Minor, 1997 ▶); data reduction: *DENZO*/*SCALEPACK*; program(s) used to solve structure: *SHELXS97* (Sheldrick, 2008 ▶); program(s) used to refine structure: *SHELXL97* (Sheldrick, 2008 ▶); molecular graphics: *SHELXTL* (Sheldrick, 2008 ▶); software used to prepare material for publication: *SHELXTL* and *publCIF* (Westrip, 2010 ▶).

## Supplementary Material

Crystal structure: contains datablock(s) I, global. DOI: 10.1107/S1600536812007799/hy2517sup1.cif

Structure factors: contains datablock(s) I. DOI: 10.1107/S1600536812007799/hy2517Isup2.hkl

Supplementary material file. DOI: 10.1107/S1600536812007799/hy2517Isup3.mol

Additional supplementary materials:  crystallographic information; 3D view; checkCIF report

## Figures and Tables

**Table 1:** Selected bond lengths (Å)

Sn1—O1	2.0965 (15)
Sn1—O1^i^	2.0866 (16)
Sn1—O2	2.0085 (15)
Sn1—O3	1.9934 (17)
Sn1—O4	2.1789 (17)
Sn1—Cl1	2.3930 (10)

Symmetry code: (i) 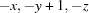.

**Table 2:** Hydrogen-bond geometry (Å, °)

*D*—H⋯*A*	*D*—H	H⋯*A*	*D*⋯*A*	*D*—H⋯*A*
O4—H13⋯O2^i^	0.78 (3)	1.94 (3)	2.696 (2)	164 (3)

Symmetry code: (i) 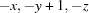.

**Fig. 1.**
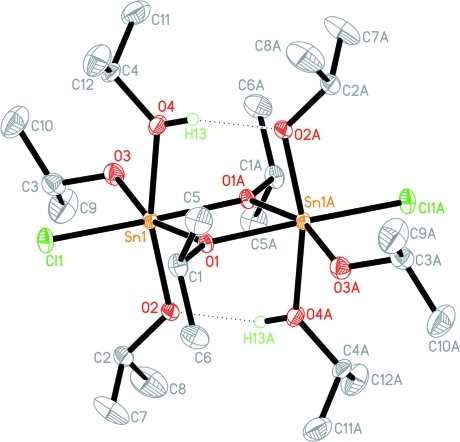
Molecular structure of the title compound. Displacement ellipsoids are shown at the 30% probability level. H atoms (except the hydroxy H atoms) have been omitted for clarity. Dotted lines denote hydrogen bonds. [Symmetry code: (A) -x, 1-y, -z.]

## supplementary crystallographic information

### Comment

The structural features of the title compound are consistent with those of other dimeric tin(IV) alkoxides, such as SnCl_3_(OR).ROH [R = Me (Sterr & Mattes, 1963) and Et (Genge *et al.*, 1996; Webster & Collins, 1974)], SnCl_3_(OCH_3_).2CH_3_OH (Reuter & Schröder, 1992), Sn(O^i^Pr)_4_.^i^PrOH (Hampden-Smith *et al.*, 1991; Reuter & Kremser, 1991), SnCl(O^i^Bu)_3_.HO^i^Bu (Reuter & Kremser, 1993), Sn(O^i^Bu)_4_.HO^i^Bu (Chandler *et al.*, 1995) and Sn_2_(CH_3_O)_2_Cl_6_(C_3_H_7_NO)_2_ (Zhang *et al.*, 2011). In all these cases, two octahedrally coordinated Sn atoms are bridged by two µ-OR groups. The molecular structure of the title compound (Fig. 1), [Sn_2_Cl_2_(µ-O^i^Pr)_2_(O^i^Pr)_4_(^i^PrOH)_2_], can be described as distorted edge-shared bi-octahedral, containing two doubly bridging isopropoxide ligands, with two terminal alkoxide ligands (one bonded to each tin) and two terminal chloride ligands in the same plane and four other ligands perpendicular to this plane (two on each metal) that are involved in hydrogen bonding. The molecule has a crystallographically imposed inversion centre. In the (RO)_2_Sn(µ-OR)_2_Sn(OR)_2_ plane, the terminal Sn—O [1.9934 (17) Å] and Sn—Cl [2.3930 (10) Å] distances are longer (Table 1), but comparable to those observed in SnCl(O^i^Bu)_3_.HO^i^Bu (Reuter & Kremser, 1993) [1.961 and 2.363 Å], while the Sn—(µ-OR) distance [2.0866 (16) Å] is analogous to those of SnCl(O^i^Bu)_3_.HO^i^Bu (2.092 Å). Perpendicular to the (RO)_2_Sn(µ-OR)_2_Sn(OR)_2_ plane, there are two isopropoxide ligands and two coordinated propan-2-ol ligands that are involved in hydrogen bonding (Table 2). The hydrogen atom was located in the final difference map. The OH-proton is located nearer to the isopropoxo group with the longest Sn—O bond [2.1789 (17) Å].

### Experimental

Acetyl chloride (0.38 g, 4.8 mmol) was added dropwise to a stirred solution of stannic alkoxide Sn(O^i^Pr)_4_.HO^i^Pr (1.99 g, 4.8 mmol) in 16 ml of anhydrous benzene at room temperature under argon using Schlenk techniques. The reaction was slightly exothermic. The reaction mixture was refluxed under stirring for one hour at 90–95°C and then allowed to reach room temperature. After three weeks, a great deal of colourless crystals were obtained (yield: about 0.76 g, 40% on tin).

### Refinement

H atom of the hydroxy group was found from a difference Fourier map and refined isotropically. H atoms on all C atoms were included in calculated positions and constrained to an ideal geometry, with C—H = 1.00 (CH) and 0.98 (CH_3_) Å and with *U*_iso_(H) = 1.2(1.5 for methyl)*U*_eq_(C). The highest residual electron density was found at 0.66 Å from O3 atom and the deepest hole at 0.91 Å from Sn1 atom.

### Crystal data

**Table:**

[Sn_2_(C_3_H_7_O)_6_Cl_2_(C_3_H_8_O)_2_]	*F*(000) = 800
*M**_r_* = 783.02	*D*_x_ = 1.489 Mg m^−^^3^
Monoclinic, *P*2_1_/*n*	Mo *K*α radiation, λ = 0.71073 Å
Hall symbol: -P 2yn	Cell parameters from 9138 reflections
*a* = 11.184 (2) Å	θ = 2.4–26.3°
*b* = 10.354 (2) Å	µ = 1.62 mm^−^^1^
*c* = 15.426 (3) Å	*T* = 173 K
β = 102.19 (3)°	Prism, colourless
*V* = 1746.0 (6) Å^3^	0.50 × 0.35 × 0.29 mm
*Z* = 2	

### Data collection

**Table:**

Nonius KappaCCD diffractometer	3539 independent reflections
Radiation source: sealed tube	3075 reflections with *I* > 2σ(*I*)
Graphite monochromator	*R*_int_ = 0.029
Detector resolution: 9 pixels mm^-1^	θ_max_ = 26.4°, θ_min_ = 2.1°
phi and ω scans	*h* = −13→12
Absorption correction: multi-scan (*DENZO*/*SCALEPACK*; Otwinowski & Minor, 1997)	*k* = −12→12
*T*_min_ = 0.498, *T*_max_ = 0.651	*l* = −19→19
16149 measured reflections	

### Refinement

**Table:**

Refinement on *F*^2^	Secondary atom site location: difference Fourier map
Least-squares matrix: full	Hydrogen site location: inferred from neighbouring sites
*R*[*F*^2^ > 2σ(*F*^2^)] = 0.021	H atoms treated by a mixture of independent and constrained refinement
*wR*(*F*^2^) = 0.045	*w* = 1/[σ^2^(*F*_o_^2^) + (0.0098*P*)^2^ + 1.9079*P*] where *P* = (*F*_o_^2^ + 2*F*_c_^2^)/3
*S* = 1.03	(Δ/σ)_max_ < 0.001
3539 reflections	Δρ_max_ = 0.45 e Å^−^^3^
175 parameters	Δρ_min_ = −0.32 e Å^−^^3^
Primary atom site location: structure-invariant direct methods	

### Special details

**Table:**

Geometry. All e.s.d.'s (except the e.s.d. in the dihedral angle between two l.s. planes) are estimated using the full covariance matrix. The cell e.s.d.'s are taken into account individually in the estimation of e.s.d.'s in distances, angles and torsion angles; correlations between e.s.d.'s in cell parameters are only used when they are defined by crystal symmetry. An approximate (isotropic) treatment of cell e.s.d.'s is used for estimating e.s.d.'s involving l.s. planes.
Refinement. Refinement of *F*^2^ against ALL reflections. The weighted *R*-factor *wR* and goodness of fit *S* are based on *F*^2^, conventional *R*-factors *R* are based on *F*, with *F* set to zero for negative *F*^2^. The threshold expression of *F*^2^ > σ(*F*^2^) is used only for calculating *R*-factors(gt) *etc*. and is not relevant to the choice of reflections for refinement. *R*-factors based on *F*^2^ are statistically about twice as large as those based on *F*, and *R*- factors based on ALL data will be even larger.

### Fractional atomic coordinates and isotropic or equivalent isotropic displacement parameters (Å^2^)

**Table:**

	*x*	*y*	*z*	*U*_iso_*/*U*_eq_	
Sn1	0.132953 (13)	0.420782 (15)	0.027075 (9)	0.02103 (5)	
Cl1	0.30397 (5)	0.44293 (6)	0.14894 (4)	0.03425 (14)	
O1	0.03597 (13)	0.57912 (14)	0.06175 (9)	0.0217 (3)	
O2	0.18898 (14)	0.53857 (16)	−0.05988 (10)	0.0292 (4)	
O3	0.17710 (15)	0.24347 (16)	−0.00562 (11)	0.0332 (4)	
O4	0.02365 (17)	0.33292 (18)	0.11269 (11)	0.0335 (4)	
C1	0.0799 (2)	0.6650 (2)	0.13774 (15)	0.0303 (5)	
H1	0.1577	0.6271	0.1723	0.036*	
C2	0.3120 (2)	0.5517 (3)	−0.07082 (17)	0.0377 (6)	
H2	0.3627	0.4813	−0.0370	0.045*	
C3	0.2933 (2)	0.1919 (3)	0.00686 (17)	0.0379 (6)	
H3	0.3543	0.2578	0.0355	0.045*	
C4	0.0516 (2)	0.2316 (2)	0.17833 (15)	0.0314 (6)	
H4	0.1384	0.2044	0.1822	0.038*	
C5	−0.0091 (3)	0.6699 (3)	0.19870 (16)	0.0439 (7)	
H5A	−0.0855	0.7101	0.1677	0.066*	
H5B	0.0262	0.7207	0.2515	0.066*	
H5C	−0.0258	0.5820	0.2165	0.066*	
C6	0.1106 (3)	0.7943 (3)	0.10514 (19)	0.0458 (7)	
H6A	0.1689	0.7836	0.0664	0.069*	
H6B	0.1471	0.8487	0.1558	0.069*	
H6C	0.0359	0.8353	0.0719	0.069*	
C7	0.3629 (3)	0.6785 (4)	−0.0357 (3)	0.0776 (12)	
H7A	0.3149	0.7483	−0.0693	0.116*	
H7B	0.4482	0.6852	−0.0417	0.116*	
H7C	0.3591	0.6857	0.0270	0.116*	
C8	0.3140 (3)	0.5378 (4)	−0.1677 (2)	0.0755 (12)	
H8A	0.2776	0.4547	−0.1895	0.113*	
H8B	0.3987	0.5413	−0.1754	0.113*	
H8C	0.2670	0.6081	−0.2012	0.113*	
C9	0.3193 (3)	0.1546 (4)	−0.0820 (2)	0.0693 (10)	
H9A	0.2590	0.0908	−0.1105	0.104*	
H9B	0.4016	0.1175	−0.0734	0.104*	
H9C	0.3142	0.2315	−0.1197	0.104*	
C10	0.3020 (3)	0.0748 (3)	0.0657 (2)	0.0683 (10)	
H10A	0.2861	0.1000	0.1234	0.102*	
H10B	0.3842	0.0376	0.0738	0.102*	
H10C	0.2414	0.0107	0.0381	0.102*	
C11	−0.0289 (3)	0.1175 (3)	0.1493 (2)	0.0602 (9)	
H11A	−0.1143	0.1411	0.1475	0.090*	
H11B	−0.0051	0.0464	0.1914	0.090*	
H11C	−0.0201	0.0903	0.0902	0.090*	
C12	0.0411 (3)	0.2839 (3)	0.26696 (18)	0.0586 (9)	
H12A	0.0952	0.3587	0.2817	0.088*	
H12B	0.0649	0.2169	0.3122	0.088*	
H12C	−0.0436	0.3101	0.2650	0.088*	
H13	−0.042 (3)	0.361 (3)	0.104 (2)	0.051 (10)*	

### Atomic displacement parameters (Å^2^)

**Table:**

	*U*^11^	*U*^22^	*U*^33^	*U*^12^	*U*^13^	*U*^23^
Sn1	0.01826 (9)	0.02480 (9)	0.01929 (8)	0.00085 (7)	0.00231 (5)	0.00071 (6)
Cl1	0.0261 (3)	0.0421 (4)	0.0292 (3)	0.0027 (3)	−0.0064 (2)	−0.0005 (3)
O1	0.0191 (8)	0.0245 (8)	0.0198 (7)	−0.0002 (7)	0.0002 (6)	−0.0041 (6)
O2	0.0200 (8)	0.0389 (10)	0.0293 (8)	−0.0023 (7)	0.0064 (7)	0.0099 (7)
O3	0.0311 (9)	0.0295 (9)	0.0367 (9)	−0.0017 (8)	0.0020 (7)	−0.0051 (7)
O4	0.0251 (10)	0.0405 (11)	0.0371 (10)	0.0094 (9)	0.0120 (8)	0.0186 (8)
C1	0.0300 (13)	0.0345 (14)	0.0236 (11)	−0.0015 (11)	−0.0009 (10)	−0.0105 (10)
C2	0.0233 (13)	0.0475 (17)	0.0444 (15)	0.0028 (12)	0.0120 (11)	0.0140 (12)
C3	0.0367 (15)	0.0325 (14)	0.0452 (15)	−0.0021 (12)	0.0101 (12)	−0.0062 (12)
C4	0.0317 (13)	0.0345 (14)	0.0291 (12)	0.0057 (11)	0.0091 (10)	0.0131 (10)
C5	0.0553 (18)	0.0507 (18)	0.0262 (13)	−0.0021 (15)	0.0098 (12)	−0.0120 (12)
C6	0.0498 (18)	0.0380 (16)	0.0489 (16)	−0.0137 (14)	0.0089 (14)	−0.0153 (13)
C7	0.050 (2)	0.090 (3)	0.096 (3)	−0.036 (2)	0.024 (2)	−0.016 (2)
C8	0.068 (2)	0.107 (3)	0.066 (2)	−0.020 (2)	0.046 (2)	−0.015 (2)
C9	0.071 (2)	0.084 (3)	0.062 (2)	0.031 (2)	0.0336 (18)	0.0088 (19)
C10	0.072 (2)	0.067 (2)	0.071 (2)	0.032 (2)	0.0261 (19)	0.0242 (19)
C11	0.073 (2)	0.0398 (17)	0.062 (2)	−0.0063 (16)	0.0021 (17)	0.0145 (15)
C12	0.075 (2)	0.070 (2)	0.0316 (15)	0.0147 (19)	0.0118 (15)	0.0076 (14)

### Geometric parameters (Å, º)

**Table:**

Sn1—O1	2.0965 (15)	C5—H5B	0.9800
Sn1—O1^i^	2.0866 (16)	C5—H5C	0.9800
Sn1—O2	2.0085 (15)	C6—H6A	0.9800
Sn1—O3	1.9934 (17)	C6—H6B	0.9800
Sn1—O4	2.1789 (17)	C6—H6C	0.9800
Sn1—Cl1	2.3930 (10)	C7—H7A	0.9800
O1—C1	1.471 (3)	C7—H7B	0.9800
O2—C2	1.428 (3)	C7—H7C	0.9800
O3—C3	1.380 (3)	C8—H8A	0.9800
O4—C4	1.445 (3)	C8—H8B	0.9800
O4—H13	0.78 (3)	C8—H8C	0.9800
C1—C6	1.496 (4)	C9—H9A	0.9800
C1—C5	1.508 (3)	C9—H9B	0.9800
C1—H1	1.0000	C9—H9C	0.9800
C2—C7	1.487 (4)	C10—H10A	0.9800
C2—C8	1.506 (4)	C10—H10B	0.9800
C2—H2	1.0000	C10—H10C	0.9800
C3—C10	1.505 (4)	C11—H11A	0.9800
C3—C9	1.510 (4)	C11—H11B	0.9800
C3—H3	1.0000	C11—H11C	0.9800
C4—C11	1.495 (4)	C12—H12A	0.9800
C4—C12	1.498 (4)	C12—H12B	0.9800
C4—H4	1.0000	C12—H12C	0.9800
C5—H5A	0.9800		
			
O3—Sn1—O2	105.15 (7)	C1—C5—H5B	109.5
O3—Sn1—O1^i^	94.16 (6)	H5A—C5—H5B	109.5
O2—Sn1—O1^i^	85.87 (6)	C1—C5—H5C	109.5
O3—Sn1—O1	162.53 (6)	H5A—C5—H5C	109.5
O2—Sn1—O1	86.94 (6)	H5B—C5—H5C	109.5
O1^i^—Sn1—O1	73.84 (6)	C1—C6—H6A	109.5
O3—Sn1—O4	88.25 (7)	C1—C6—H6B	109.5
O2—Sn1—O4	162.24 (7)	H6A—C6—H6B	109.5
O1^i^—Sn1—O4	81.50 (6)	C1—C6—H6C	109.5
O1—Sn1—O4	77.60 (7)	H6A—C6—H6C	109.5
O3—Sn1—Cl1	95.02 (5)	H6B—C6—H6C	109.5
O2—Sn1—Cl1	98.98 (5)	C2—C7—H7A	109.5
O1^i^—Sn1—Cl1	168.11 (4)	C2—C7—H7B	109.5
O1—Sn1—Cl1	95.47 (4)	H7A—C7—H7B	109.5
O4—Sn1—Cl1	91.23 (5)	C2—C7—H7C	109.5
C1—O1—Sn1^i^	128.84 (13)	H7A—C7—H7C	109.5
C1—O1—Sn1	124.90 (13)	H7B—C7—H7C	109.5
Sn1^i^—O1—Sn1	106.16 (6)	C2—C8—H8A	109.5
C2—O2—Sn1	125.52 (14)	C2—C8—H8B	109.5
C3—O3—Sn1	126.66 (15)	H8A—C8—H8B	109.5
C4—O4—Sn1	131.40 (15)	C2—C8—H8C	109.5
C4—O4—H13	116 (2)	H8A—C8—H8C	109.5
Sn1—O4—H13	112 (2)	H8B—C8—H8C	109.5
O1—C1—C6	109.58 (19)	C3—C9—H9A	109.5
O1—C1—C5	111.34 (19)	C3—C9—H9B	109.5
C6—C1—C5	114.1 (2)	H9A—C9—H9B	109.5
O1—C1—H1	107.2	C3—C9—H9C	109.5
C6—C1—H1	107.2	H9A—C9—H9C	109.5
C5—C1—H1	107.2	H9B—C9—H9C	109.5
O2—C2—C7	110.2 (2)	C3—C10—H10A	109.5
O2—C2—C8	108.9 (2)	C3—C10—H10B	109.5
C7—C2—C8	111.2 (3)	H10A—C10—H10B	109.5
O2—C2—H2	108.8	C3—C10—H10C	109.5
C7—C2—H2	108.8	H10A—C10—H10C	109.5
C8—C2—H2	108.8	H10B—C10—H10C	109.5
O3—C3—C10	109.6 (2)	C4—C11—H11A	109.5
O3—C3—C9	109.2 (2)	C4—C11—H11B	109.5
C10—C3—C9	109.9 (3)	H11A—C11—H11B	109.5
O3—C3—H3	109.4	C4—C11—H11C	109.5
C10—C3—H3	109.4	H11A—C11—H11C	109.5
C9—C3—H3	109.4	H11B—C11—H11C	109.5
O4—C4—C11	109.6 (2)	C4—C12—H12A	109.5
O4—C4—C12	109.3 (2)	C4—C12—H12B	109.5
C11—C4—C12	113.6 (2)	H12A—C12—H12B	109.5
O4—C4—H4	108.1	C4—C12—H12C	109.5
C11—C4—H4	108.1	H12A—C12—H12C	109.5
C12—C4—H4	108.1	H12B—C12—H12C	109.5
C1—C5—H5A	109.5		
			
O3—Sn1—O1—C1	128.6 (2)	O4—Sn1—O3—C3	−120.29 (19)
O2—Sn1—O1—C1	−96.80 (16)	Cl1—Sn1—O3—C3	−29.20 (19)
O1^i^—Sn1—O1—C1	176.62 (19)	O3—Sn1—O4—C4	48.2 (2)
O4—Sn1—O1—C1	92.01 (16)	O2—Sn1—O4—C4	−172.1 (2)
Cl1—Sn1—O1—C1	1.94 (16)	O1^i^—Sn1—O4—C4	142.7 (2)
O3—Sn1—O1—Sn1^i^	−48.0 (2)	O1—Sn1—O4—C4	−142.1 (2)
O2—Sn1—O1—Sn1^i^	86.58 (7)	Cl1—Sn1—O4—C4	−46.7 (2)
O1^i^—Sn1—O1—Sn1^i^	0.0	Sn1^i^—O1—C1—C6	−74.9 (2)
O4—Sn1—O1—Sn1^i^	−84.61 (7)	Sn1—O1—C1—C6	109.3 (2)
Cl1—Sn1—O1—Sn1^i^	−174.67 (5)	Sn1^i^—O1—C1—C5	52.2 (3)
O3—Sn1—O2—C2	−60.20 (19)	Sn1—O1—C1—C5	−123.58 (19)
O1^i^—Sn1—O2—C2	−153.40 (19)	Sn1—O2—C2—C7	−108.4 (3)
O1—Sn1—O2—C2	132.60 (19)	Sn1—O2—C2—C8	129.3 (2)
O4—Sn1—O2—C2	161.9 (2)	Sn1—O3—C3—C10	119.9 (2)
Cl1—Sn1—O2—C2	37.53 (19)	Sn1—O3—C3—C9	−119.6 (2)
O2—Sn1—O3—C3	71.53 (19)	Sn1—O4—C4—C11	−115.7 (2)
O1^i^—Sn1—O3—C3	158.37 (19)	Sn1—O4—C4—C12	119.3 (2)
O1—Sn1—O3—C3	−155.9 (2)		

Symmetry code: (i) −*x*, −*y*+1, −*z*.

### Hydrogen-bond geometry (Å, º)

**Table:**

*D*—H···*A*	*D*—H	H···*A*	*D*···*A*	*D*—H···*A*
O4—H13···O2^i^	0.78 (3)	1.94 (3)	2.696 (2)	164 (3)

Symmetry code: (i) −*x*, −*y*+1, −*z*.

## References

[bb1] Chandler, C. D., Caruso, J., Hampden-Smith, M. J. & Rheingold, A. (1995). *Polyhedron*, **14**, 2491–2497.

[bb2] Genge, A. R. J., Levason, W. & Reid, G. (1996). *Acta Cryst.* C**52**, 1666–1668.

[bb3] Hampden-Smith, M. J., Wark, T. A., Rheingold, A. & Huffman, J. C. (1991). *Can. J. Chem.* **69**, 121–129.

[bb4] Mehrotra, R. C. & Gupta, V. D. (1966). *J. Indian Chem. Soc.* **43**, 155–160.

[bb5] Nonius (1998). *COLLECT* Nonius BV, Delft, The Netherlands.

[bb6] Otwinowski, Z. & Minor, W. (1997). *Methods in Enzymology*, Vol. 276, *Macromolecular Crystallography*, Part A, edited by C. W. Carter Jr & R. M. Sweet, pp. 307–326. New York: Academic Press.

[bb7] Reuter, H. & Kremser, M. (1991). *Z. Anorg. Allg. Chem.* **599–600**, 271–280.

[bb8] Reuter, H. & Kremser, M. (1993). *Z. Kristallogr.* **203**, 158–160.

[bb9] Reuter, H. & Schröder, D. (1992). *Acta Cryst.* C**48**, 1112–1114.

[bb10] Sheldrick, G. M. (2008). *Acta Cryst.* A**64**, 112–122. 10.1107/S0108767307043930 18156677

[bb11] Sterr, G. & Mattes, R. (1963). *Z. Anorg. Allg. Chem.* **322**, 319–325.

[bb12] Vaartstra, B. A., Huffman, J. C., Gradeff, P. S., Hubert-Pfalzgraf, L. G., Daran, J. C., Parraud, S., Yunlu, K. & Caulton, K. G. (1990). *Inorg. Chem.* **29**, 3126–3131.

[bb13] Webster, M. & Collins, P. H. (1974). *Inorg. Chim. Acta*, **9**, 157–160.

[bb14] Westrip, S. P. (2010). *J. Appl. Cryst.* **43**, 920–925.

[bb15] Zhang, Q., Yin, H. & Wang, D. (2011). *Acta Cryst.* E**67**, m146. 10.1107/S1600536810053997 PMC3051740 21522830

